# Transforming Global Health Communications During the COVID-19 Pandemic: International Partner Perspectives

**DOI:** 10.5334/aogh.3531

**Published:** 2022-02-03

**Authors:** Mitra Sadigh, Swapnil Parve, Jamidah Nakato, Hamidah Babirye Nsereko, Majid Sadigh

**Affiliations:** 1 Nuvance Health/University of Vermont Larner College of Medicine Global Health Program, CT and VT, US; 2 Kazan State Medical University, Kazan, Russia; 3 Datta Meghe Institute of Medical Sciences (Deemed to be University), Sawangi Meghe, IN; 4 Makerere University, Kampala, UG

## Abstract

**Background:**

With the COVID-19 pandemic restricting travel, global health programs are faced with the challenge of bidirectionally supporting students, partners, and communities in new ways. Though other global health programs have—to the best of our knowledge—temporarily frozen, we at the Nuvance Health/University of Vermont Larner College of Medicine Global Health Program have carried forward by transforming our communications through launching a COVID-19 Resources Page with bi-weekly article summaries, redirecting our monthly eMagazine and weekly blog to pandemic themes, and staying in constant communication with our partners around the world.

**Objective:**

To investigate the extent to which our program’s published content shifted in sync with the COVID-19 pandemic, as well as our international partners’ perception of the COVID-19 resource center, eMagazine, and blog in terms of relevance, representation, and utility.

**Methods:**

A survey consisting of quantitative questions and open-ended response questions was allocated along the following themes: (1) eMagazine; (2) Global Health Diaries blog; (3) COVID-19 Resource Center including article summaries; and (4) communications. It was sent to 34 leaders in our partner sites across nine countries—Botswana, China, the Dominican Republic, India, Thailand, Russia, Uganda, Vietnam, and Zimbabwe—and filled out by 31.

**Findings:**

Survey results revealed overwhelmingly positive feedback from our global health partners who reported frequently using our COVID-19 resources, often as first-line information about the pandemic; feeling emotional support through our communications; enjoying content in our eMagazine and blog; and finding fair representation in our published content. Our global health program is more deeply connected than ever.

**Conclusions:**

Though global health programs seemingly have their hands tied, we are only beginning to imagine the breadth of new avenues for connectivity, learning, and sharing. We must all be creative about staying connected. There are avenues for global health advocacy yet to be discovered.

## Background

The COVID-19 pandemic has proved to be an ongoing, polymorphous crisis across all health and safety lines from public to mental, political to domestic, economic to social. Global health advocates have long been concerned about the imminent disasters lurking at the cross-section of poor public health infrastructure, increased globalization, human disregard for the precarious curtain separating us from Earth’s wilderness, and viruses that abide by no geographic bounds. Named by the United Nations Development Programme as the “defining global health crisis of our time [1],” global health programs around the world have heard the call to arms.

But with borders closed, travel restricted, and health risks abound, global health programs can no longer participate in field work and exchange of students or personnel across oceans. We cannot share physical space with our international partners, nor they with us. We cannot help care for each other’s patients or mentor one another’s students. Our exchange of stories, sentiments, knowledge, and reassurance is limited to emails and unstable phone connections, the voice on the other end likely to drop at any moment. With movement severely limited, global health programs are faced with the challenge of bidirectionally supporting students, partners, and communities in new ways.

The Nuvance Health/University of Vermont Larner of College of Medicine Global Health Program (NH/UVMLCOM GHP) supported our international partner faculty and leaders for a total of 209 weeks in 2019, for visits as well as our Global Health Scholars Program, a capacity building initiative whereby we host international partners for two to five months of training in clinical, education, and research skills. Meanwhile our international partners supported our students, residents, fellows, and faculty for a total of 395 weeks, mostly for the six-week global health elective, through which 65 medical students from the University of Vermont, American University of the Caribbean, and Ross University School of Medicine rotated. We also hosted the inaugural NH/UVMLCOM GHP conference and celebration, attended by our international partner leaders as well as many domestic partners and community members.

Needless to say, our activities since January 2020 have in no way resembled those of the past. At the onset of the pandemic, our Global Health Scholars returned home from our domestic hosting sites early, as did our medical students from their global health electives. Conferences and presentations, plans to host our partners, and our annual Global Health Day celebration were all canceled.

### Swift-Shifting Our Activities in Response to the COVID-19 Pandemic

With all of our regular activities canceled and curtailed, we were left with the task of discerning our role during the pandemic. Though a significant number of global health programs have temporarily frozen, we at the NH/UVMLCOM GHP have carried forward. With two-week virtual global health elective programs running through the end of this pandemic’s second summer—and a more comprehensive global health program as well as meaningful virtual capacity-building platforms impending—we are finding creative ways of swift-shifting into pandemic-era global health education in order to stay connected to our students. Recently launched virtual forums with our partners discussing “hot topics,” followed by Q&A sessions with other partners, faculty, students, and community members have allowed us to stay connected to our partners and communities, while a special video series brings viewers from a Ugandan hospital to patients’ homes for a discussion of social, cultural, and environmental health factors. Topics are centered on the impact of COVID-19 on communities, from clinical treatment and management to public health and economic perspectives.

### Amplifying Support to Our 21 Partner Institutions in 9 Countries Across 4 Continents

At the time of SARS-COV-2’s early rumblings when the virus had yet to reach the borders of many nations, our international partners expressed concern over the scarcity of reliable resources to help them prepare for the pandemic. Although many major academic [2] and journalistic [3] publishers have removed the paywall on COVID-19 resources, healthcare workers still face significant challenges accessing them, from lower internet penetration and unstable connections [4,5,6] to insufficient analytical tools with which to review, distill, and critically evaluate scientific articles—skills that our Global Health Program has trained a significant number of healthcare workers in over the last 30 years by developing and implementing Evidence-Based Medicine (EBM) courses worldwide [7] — to lack of time, emotional bandwidth, and human resources.

In an effort to continue growing our commitment to our partners and do our part in helping curb the COVID-19 pandemic [8], we amplified support to our 16 main partner institutions, in nine countries across four continents as well as five in the U.S., utilizing four avenues: the NH/UVMLCOM GHP COVID-19 Resource Center, our program’s eMagazine, our blog titled *Global Health Diaries*, and frequent communication.

#### COVID-19 Resource Center

We swiftly launched the COVID-19 Resource Center, a robust site featuring descriptions of and links to some of the most trusted scientific and evidence-based sources: *The Journal of the American Medical Association (JAMA), The New England Journal of Medicine (NEJM)*, The Centers for Disease Control and Prevention (CDC), The University of Washington (UW) Medicine COVID-19 Resource Site, the Report of the World Health Organization (WHO)-China Joint Mission on Coronavirus Disease 2019 (COVID-19), The Q&A on Coronavirus – COVID-19 with WHO’s Dr. Maria Van Kerkhove, and WHO Press Briefing Videos and Audio Clips. Our COVID-19 Resource Center also consists of a compilation of summaries of COVID-19-related articles in the most prestigious journals including *NEJM*, the *Lancet* Family of Journals, *JAMA Network, Nature*, and *Science*, published bi-weekly.

Our primary goal has been to gather, review, distill, and deliver the latest COVID-19 articles to our partners in a timely fashion via emails and instant messengers while hosting these summaries on our COVID-19 Resource Center. Each summary is 300–3500 words, in addition to tables and figures from the articles, with 3060 articles having been summarized overall from March 2020 to June 2021. A collaboration among one of the program’s infectious disease specialists who chose and summarized the articles, the communications editor who formatted them, and the media director who transformed the summaries into an aesthetically pleasing mobile-friendly version. These summaries are without commentary to allow readers to interpret findings for themselves. The link was shared on several institutional websites, and emailed to the master list bi-weekly. We also sent daily country-specific articles to our partners in corresponding countries. On a greater scale, these activities were carried out to help combat the growing COVID-19 “disinfodemic” [9,10] which has not only created an atmosphere of bewilderment but has exacerbated the digital divide by hindering web connectivity [11], particularly in the Global South [12].

#### eMagazine and Global Health Diaries blog

The content of our eMagazine traditionally showcased diverse sections including perspective pieces and reflection, as well as special sections such as “Global Health and the Arts,” “Article of the Month,” “Sexual Harassment and Violence Across Cultural Settings.” The monthly publication is geared toward all program members, as well as anyone who is interested in a wide lens of global health topics. It is difficult to gauge exactly how many people the eMagazine reaches, as the roughly 600 contacts on the master email list circulate it widely among their own institutions and communities. We are hoping to turn the eMagazine into an eJournal with an editor and editorial board. Meanwhile, our *Global Health Diaries* blog with weekly posts historically centered on our program members’ global health stories and experiences and featured special monthly series such as “Ethical Dilemmas in Global Health,” “Challenging Moments in Global Health,” and a student collection titled “On the Global Health Pathway.”

Both platforms were shifted toward the COVID-19 pandemic, and now serve as podiums for the pandemic-related stories, views, triumphs, and challenges of our partners, colleagues, and friends around the globe. Since the start of the pandemic, *Global Health Diaries* has been hosting a narrative series titled “In the World With COVID-19” which shares our partners’ heartfelt stories while our eMagazine currently contains exclusively COVID-19-related content, from perspective pieces representing our international partners to inspiring works of art to help cultivate hope during these trying times. In the year 2020, *Global Health Diaries* had 6 342 visitors from 102 different countries.

Both publications are a collaborative effort among the program director and communications editor who select and edit pieces, the media editor who creates and publishes the visual layout, and our many contributors. Contributors include students, residents, faculty, and administrators from our home and partner institutions in nine countries alike, as well as friends of the program. We are proactive in asking partners from all sides to reflect on certain themes, from their communities’ response to COVID-19 vaccination to the challenges of obtaining an education as a woman in the Global South. Ideas often also come from our partners, whose creative endeavors we are committed to supporting by providing a platform for their work.

We believe in building true partnerships in which leaders from all sites feel that they are co-owners of the program. Our many alumni—international colleagues who have spent time with us at UVMLCOM and Nuvance Health and affiliated hospitals, as well as domestic students and residents who completed the GHP—are frequent contributors, as are scholarship recipients. Further, friendship nurtures a desire to share in a way that a business relationship may not—a feature of our program that we believe creates an environment in which we are never short of stories to publish.

A greater collective has emerged through the course of the pandemic, through which readers want to follow and connect with others’ stories while adding their own to the growing narrative. It is through the solidarity born from sharing stories and experiences that our global health community has remained connected during these continuing difficult times.

Global health programs—and truly, all programs and people everywhere—have been called to reevaluate what support and connectivity look like. Though the pandemic has forced us to shapeshift our activities, we believe there are means of continuing our commitments even from across the world. Our hope is that readers may learn something from the experiences we have had thus far, and perhaps be encouraged toward their own ideas for creative support and connectivity.

We investigated the extent to which our program’s published content shifted in sync with the COVID-19 pandemic, as well as our international partners’ perception of the COVID-19 Resource Center, eMagazine, and *Global Health Diaries* in terms of relevance, representation, and utility.

## Methods

The survey consisted of 12 quantitative questions, including linear scale and multiple check-box formats, and four open-ended response questions, all allocated along the following themes: (1) eMagazine; (2) *Global Health Diaries*; (3) COVID-19 Resource Center including article summaries; and (4) communications. It was sent to 31 leaders in our partner sites across nine countries—Botswana, China, the Dominican Republic, India, Thailand, Russia, Uganda, Vietnam, and Zimbabwe. Because this study was not related to patients or identifiable patient data, IRB approval was not requested.

We analyzed every eMagazine publication and *Global Health Diaries* post from January 2020 to January 2021 to assess the degree to which the content was repurposed to focus on COVID-19 and the perspectives of our international partners. Frequency tables were derived to ascertain the numbers of international and local U.S. perspectives on the COVID-19 pandemic.

## Results

The survey was filled out by 29 out of 31 recipients, yielding a 94% response rate. An overwhelming majority of surveyees agreed that the NH/UVMLCOM GHP eMagazine and *Global Health Diaries* tuned in sync with the demands that arose from the COVID-19 pandemic, reflects (-ed) their challenges, and served as podiums for their experiences and interests.

The NH/UVMLCOM GHP COVID-19 Resource Center was reported as the second most preferred source of COVID-19 information for most of our international partners, after government reports and recommendations. Most surveyees reported “almost always” referring to our COVID-19 Resource Center and article summaries. Moreover, frequent and consistent communication with our international partners since the pandemic’s onset was attested to by most surveyees, with email reported as the most desired form of communication followed by instant messengers such as WhatsApp, Viber, Telegram, iMessage, Skype, etc.

**Figures 1–3:**
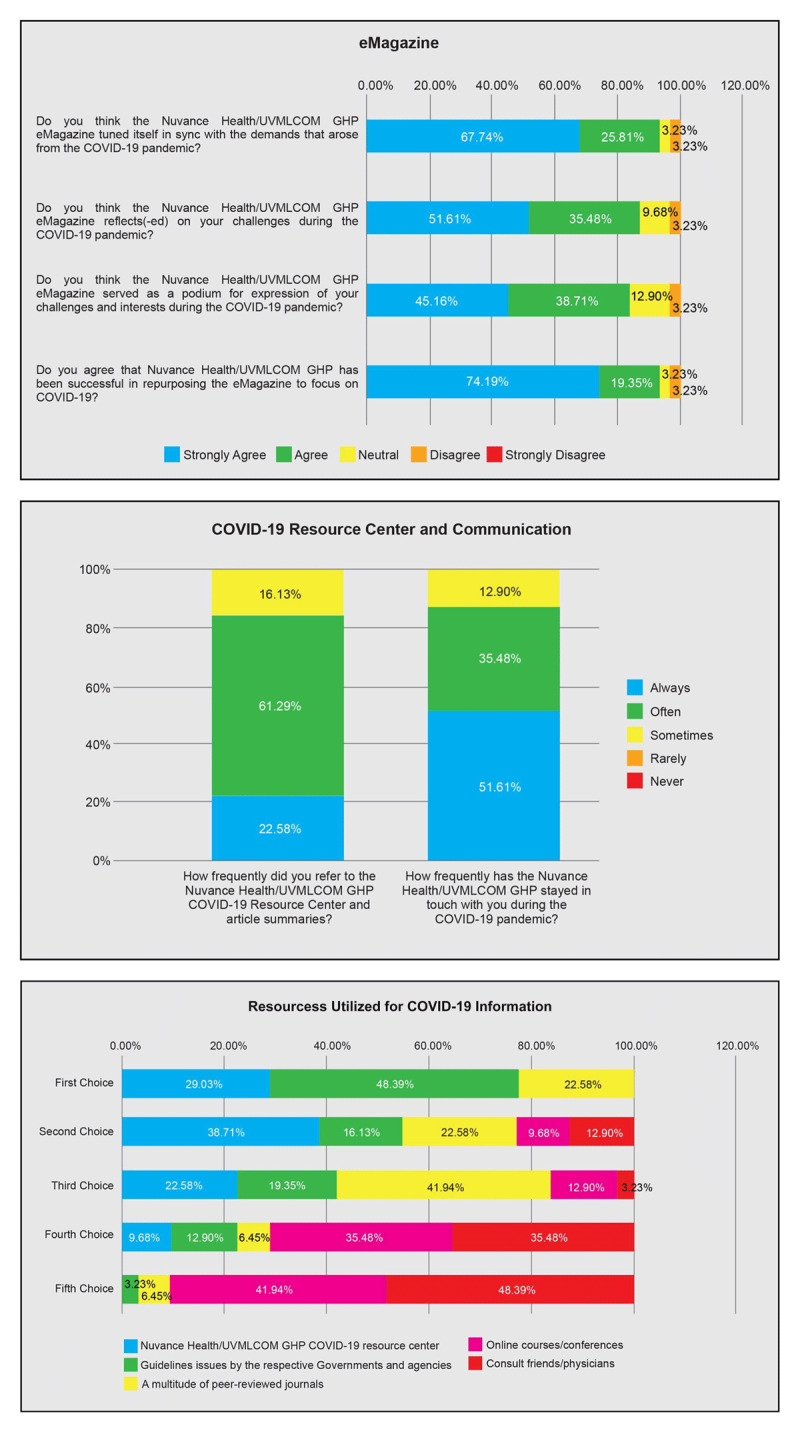
Surveyees’ perceptions of the eMagazine in focusing on the COVID-19 pandemic and reflecting their experiences. Surveyees’ usage of the COVID-19 Resource Center and perception of communication with the NH/UVMLCOM GHP. Resources surveyees referred to for COVID-19 information.

### eMagazine

Feedback regarding eMagazine content was entirely positive, with surveyees applauding it as “applicable,” “useful,” “on target,” “informative,” “having wide coverage,” “valuable,” “concise,” helpful,” “timely,” and “critical.”

Surveyees spoke to its relevance, importance, and interest:

*“Excellent resource to keep informed about the pandemic and its impact all around the world.”*

*“The content of the eMagazine is versatile. The opinions of various experts on the pandemic and the experience of some countries in fighting the infection are also interesting.”*

As relaying pandemic-related emotional challenges:

*“The personal stories of how caregivers felt caring for patients who end up dying alone was very touching.”*

Preparing readers for pandemic-related emotional challenges:

*“The eMagazine helped those of us who are still to see COVID cases prepare emotionally for that eventuality.”*

And helping encourage evidence-based COVID-related practices:

*“I learned guidelines and recommendations which served my clinical practices in Vietnam.”*

*“The e-Magazine provides important epidemic data and information that can be useful in clinical practice.”*

*“It provides important epidemic data and information that can be useful in clinical practice.”*

Suggestions were categorized as follows: content representing a greater scope of nations (4); greater COVID-19-related clinical material, including diagnostics, treatment, management, and specific case discussions (4); a greater scope of disciplines, including the views of non-clinical stakeholders and the holistic approach to health rather than high medical specialization (2); and greater resources pertaining to medical education and global health activities in the context of the pandemic (2).

### Global Health Diaries

All responses were overwhelmingly positive in regard to *Global Health Diaries* since the onset of the COVID-19 pandemic, with content described as “vibrant,” “relevant,” “useful,” “helpful,” “informative,” “wonderful,” “excellent,” and “expanding our horizons and learning about problems in the world.”

Surveyees spoke to its role in keeping the program members connected:

*“It brings the teams together and keeps the collaboration alive.”*

*“It was very useful to (1) get first-hand experience of fighting the pandemic in other countries, their challenges and successes; (2) realize the strengths which could be implemented in our own practice; and (3) realize that doctors in other countries share the same (or quite similar) concerns and challenges which facilitates overcoming them.”*

To its role in helping readers in a time of crisis:

*“It brings hope to all people because it addresses challenges faced.”*

And to its variety and representation:

*“The blog provides information on a range of pandemic-related issues: from epidemiology to psychology.”*

*“It is fully balanced and caters to all partners including students.”*

The three standalone suggestions include virtual dialogues, conferences, or webinars to encourage further engagement; greater representation of international partner student perspectives; and more equal representation of all of our partners.

The very first COVID-19-related piece was posted on *Global Health Diaries* on March 8, four days after the COVID-19 crisis was officially announced as a pandemic.

### NH/UVMLCOM GHP COVID-19 Resource Center, Including Article Summaries

All responses all were incredibly positive, citing the Resource Center as “up-to-date,” “reliable,” “concise,” “useful,” “excellent,” “on point,” “easy to navigate,” “comprehensive,” “time-saving,” “widely referenced” and as presenting “the newest information from the best sources.” Highlights include:

*“Thank you very much to everyone who works on this project and helps people get reliable information (in the context of the ‘fake’ pandemic).”*

*“This is a fairly promising form of reporting globally.”*

*“They cause relief out of curiosity and fear.”*

*“It is a wonderful digest of articles that is very helpful for keeping up with the latest news about COVID-19, spread of the virus, and experience of fighting it, especially under time constraints during such a difficult period.”*

Suggestions included covering partner country-specific COVID-19 information (3) and making the summaries shorter and more concise (1).

### Circulation of the NH/UVMLCOM GHP Covid-19 Resource Center, Including Article Summaries

We explored the extent to which our partners were circulating the NH/UVMLCOM GHP COVID-19 Resource Center and article summaries. Because the center’s platform lacks a tracking mechanism, and the link and its contents have been shared across countless platforms, we can only estimate the number of visitors to our COVID-19 Resource Center. To provide a tentative estimate on circulation numbers, we consider “direct reach,” delivery through means such as emails and postings on WhatsApp groups and Telegram Channels, and “indirect reach,” delivery through means such as posting on webpages and social media accounts, both of which were utilized in disseminating the Resource Center. The link has been shared on institutional pages in several countries, and program members report having shared it with anywhere from 20 to over 400 personal contacts, with select article summaries having been translated into Farsi, Russian, and Vietnamese. The center is also hyperlinked on our *Global Health Diaries* blog page, which had over 6,000 viewers in 2020, in addition to the eMagazine and program website. Based on both direct and indirect reach, we estimate that our summaries were received by more than 10 000 people across the USA, our partner institutions, and beyond.

*There has been so much pressure on some of us involved in guideline development to keep responding to articles/adopting the findings of these few studies! Thanks for keeping us informed. I have also shared with our colleagues here and our postgraduate students who are usually tempted to adopt new medicines as and when they arise! I am rather old fashioned and tend to approach these recommendations with my “essential medicine concept” lens and hence allow myself to take a deep breath in before adopting new/old medicines!*

#### Professor Chiratidzo Ellen Ndhlovu, MD, Site Director of the NH/UVMLCOM GHP at the University of Zimbabwe Faculty of Medicine and Health Sciences

Quotation shared with permission.

### Contributions of Our International Partners to the NH/UVMLCOM GHP During the COVID-19 Pandemic

A majority of surveyees reported having contributed to the NH/UVMLCOM GHP during the COVID-19 pandemic. This took the form of sharing suggestions, information, and experiences including translating and sharing the seventh edition of China’s COVID-19 Prevention and Control Guidelines, writing reflections and articles for *Global Health Diaries* and the eMagazine, and sharing resources distributed by the GHP with colleagues, faculty, and students, including translation. Other activities included communicating via emails and calls, commenting on distributed materials, and participating in student engagement, teaching, and mentorship.

## Discussion

Survey results indicate positive reception of our COVID-19 resources page, eMagazine, and *Global Health Diaries* by all surveyees with all three platforms having been perceived as helpful, relevant, and reflective of our international partners’ perspectives. The results also indicate that the content of the eMagazine and *Global Health Diaries* swift-shifted to center on COVID-19-related materials. The increasing amount of COVID-19-related material in our publications aligns with increased numbers of COVID-19 cases and, as a natural extension, increased numbers of people talking about the pandemic. Meanwhile, the seesawing of content to and from international and U.S. perspectives likely reflects the ebb and flow of evolving hot-zone areas.

### COVID-19 Resource Center

We conjecture that the article summaries have been positively received and appreciated in large part because they are, to the best of our knowledge, comprehensive compilations of articles published in the most major journals in a form not found elsewhere. With a table of contents categorized by topic, it is also convenient and simple to navigate.

Per the suggestion to provide partner country-specific COVID-19 information, we are limited to those countries represented in our sources. With the help of our international partners, we could compile relevant articles to be summarized and shared. Per the suggestion to make summaries shorter and more concise, we fear that creating “takeaway points” may infuse our biases into the summaries, but we have highlighted readers’ options of skimming for italic text and figures in order to shorten reading time.

### Global Health Diaries

The results suggest that the *Global Health Diaries* content has helped program members feel connected through each other’s stories, and provided an emotional outlet during the COVID-19 pandemic. From the vulnerable struggle of an intensive care unit physician in Florida to a Ugandan doctor’s advice about how Global North healthcare workers can manage scarcity and tragedy; a physician’s commentary on a Zimbabwean artist’s visual rendition of physical distancing (Lin Barrie’s “To Touch… Or Not to Touch”) to a Vietnamese physician sharing the story of successful confinement, *Global Health Diaries* is rich in range, depth, and representation.

Though we appreciate the suggestion of furthering engagement through virtual dialogues, conferences, or webinars, the aforementioned digital divide and heavy web traffic present major barriers. We could begin by encouraging more comments directly on blog posts to spark conversation. We also appreciate the two suggestions of representing perspectives from international partner students and of our international partner sites equally. Because we post all pieces shared with us, lack of representation indicates an absence of submissions from respective sources which may be attributed to busy schedules and/or language barriers. In response, we would like to create a system of encouraging our less frequently-published partners to submit reflections to be posted and shared. We would be delighted to showcase the perspectives of each and every one of our partners and members.

### eMagazine

Results suggest that the eMagazine content similarly relayed the emotional experience of our partners around the world, and is perceived to have covered a broad range of topics. Per the suggestions, adding content representing a broader scope of nations, including perspectives of the holistic approach to health as well as non-clinical stakeholders; addressing resources for medical education and global health activities in the midst of the pandemic; and sharing COVID-19-related clinical materials would all add to the quality of our content.

## Conclusion

Despite our Global Health Program and members facing numerous challenges amidst the COVID-19 pandemic, we understand and have taken to heart the vitality of connectivity. We quickly repurposed our activities to support and stay in commune with our students and partners. Our communications platform has risen as a beacon of exchange for information, stories, support, and hope. We have counseled each other on difficult days and inspired each other on resilient ones. We have shared ideas as we observed, and imparted wisdom as we learned. This time has deepened and broadened our connection. Today, we and our partners are closer than ever despite the disruption the pandemic has had on many of our partners’ other longitudinal global health partnerships.

Though it may appear that global health programs have their hands tied, we are only beginning to imagine the breadth of new avenues for connectivity, learning, and sharing. Our message to global health programs around the world is this: Be creative about staying connected and harness the power of communications. There are avenues for global health advocacy yet to be discovered.

## Author Information

All authors had access to data and a role in writing the manuscript.
